# Improved prehospital triage for acute cardiac care: results from HART-c, a multicentre prospective study

**DOI:** 10.1007/s12471-023-01766-3

**Published:** 2023-03-29

**Authors:** Enrico R. de Koning, Saskia L. M. A. Beeres, Jan Bosch, Barbra E. Backus, Wouter J. Tietge, Reza Alizadeh Dehnavi, Rolf H. H. Groenwold, Allena M. Silvius, Pepijn T. S. van Lierop, J. Wouter Jukema, Martin J. Schalij, Mark J. Boogers

**Affiliations:** 1 Department of Cardiology, Leiden University Medical Centre, Leiden, The Netherlands; 2 Research and Development, Regional Ambulance Service Hollands-Midden (RAVHM), Leiden, The Netherlands; 3 Department of Emergency Medicine, Leiden University Medical Centre, Leiden, The Netherlands; 4 Department of Cardiology, Alrijne Hospital, Leiderdorp, The Netherlands; 5 Department of Cardiology, Groene Hart Hospital, Gouda, The Netherlands; 6 Department of Clinical Epidemiology, Leiden University Medical Centre, Leiden, The Netherlands; 7 Biomedical Data Sciences, Leiden University Medical Centre, Leiden, The Netherlands; 8 Department of Public Health and General Practice, Leiden University Medical Centre, Leiden, The Netherlands

**Keywords:** Prehospital triage, Telemedicine, Hollands-Midden Acute Regional Triage—cardiology

## Abstract

**Background:**

Cardiac symptoms are one of the most prevalent reasons for emergency department visits. However, over 80% of patients with such symptoms are sent home after acute cardiovascular disease has been ruled out.

**Objective:**

The Hollands-Midden Acute Regional Triage—cardiology (HART-c) study aimed to investigate whether a novel prehospital triage method, combining prehospital and hospital data with expert consultation, could increase the number of patients who could safely stay at home after emergency medical service (EMS) consultation.

**Methods:**

The triage method combined prehospital EMS data, such as electrocardiographic and vital parameters in real time, and data from regional hospitals (including previous medical records and admission capacity) with expert consultation. During the 6‑month intervention and control periods 1536 and 1376 patients, respectively, were consulted by the EMS. The primary endpoint was the percentage change of patients who could stay at home after EMS consultation.

**Results:**

The novel triage method led to a significant increase in patients who could safely stay at home, 11.8% in the intervention group versus 5.9% in the control group: odds ratio 2.31 (95% confidence interval (CI) 1.74–3.05). Of 181 patients staying at home, only 1 (< 1%) was later diagnosed with ACS; no patients died. Furthermore the number of interhospital transfers decreased: relative risk 0.81 (95% CI 0.67–0.97).

**Conclusion:**

The HART‑c triage method led to a significant decrease in interhospital transfers and an increase in patients with cardiac symptoms who could safely stay at home. The presented method thereby reduced overcrowding and, if implemented throughout the country and for other medical specialties, could potentially reduce the number of cardiac and non-cardiac hospital visits even further.

**Supplementary Information:**

The online version of this article (10.1007/s12471-023-01766-3) contains supplementary material, which is available to authorized users.

## What’s new?

- A novel prehospital triage platform for cardiologists and nurse paramedics, combining prehospital and hospital data, was developed.
- There was a significant increase in the number of patients left at home after consultation with a nurse paramedic and a cardiologist.
- A decrease in interhospital transfers resulted.
- The rate of major adverse cardiovascular events in patients left at home was low (< 1%).

## Introduction

Cardiac symptoms are one of the most prevalent reasons for emergency department (ED) visits [1]. Interestingly, the vast majority of patients with cardiac symptoms are sent home after acute cardiovascular disease has been ruled out. Previous studies showed that up to 80% of all patients with chest pain do not have an acute coronary syndrome (ACS) [2–4]. This situation calls for improvement, especially in an era in which the Dutch healthcare system is increasingly under pressure.

Overcrowding of EDs leads to worse patient outcomes, increased healthcare costs and dissatisfied patients and healthcare workers [5–8]. From a cardiologist’s perspective, previous attempts to reduce overcrowding mostly focused on rapid risk stratification to rule out ACS with the development of risk scores, including the frequently used HEART (History, Electrocardiogram, Age, Risk factors, Troponin) score [9, 10]. These risk scores, however, do not address the root cause of overcrowding, as patients are still required to visit the ED. Accordingly, attention shifted from in-hospital triage towards prehospital triage. The recent FamouS Triage [11] and ARTICA [12] studies focused on prehospital risk stratification. These studies bring progress, but focus only on chest pain patients, whereas patients with other cardiac symptoms also contribute considerably to ED overcrowding. Improved prehospital triage of all cardiac patients could potentially reduce the number of unnecessary ED visits and contribute to effectively combatting overcrowding throughout the Netherlands.

To improve prehospital triage for all patients with cardiac symptoms a comprehensive novel triage method was developed, entitled Hollands-Midden Acute Regional Triage—cardiology (HART-c). This triage method combines prehospital real-time emergency medical services (EMS) data with hospital data and direct consultation of a cardiologist with insight into the triage platform. The primary aim of the HART‑c study was to investigate whether this novel triage method could increase the number of patients who could safely stay at home.

## Methods

### Study design

The HART‑c study was a multicentre prospective cohort study with a historical control group. The intervention group comprised adult patients visited by the EMS in the Dutch region Hollands-Midden, for symptoms of suspected cardiac origin during a 6-month period, between 9 September 2019 and 6 March 2020. The historical control period was 6 months in the previous year, between 9 September 2018 and 6 March 2019. Full details of the study protocol were published previously [13].

### Intervention group—novel triage method

During the intervention period, patients presenting to the regional EMS with symptoms of suspected cardiac origin received standard clinical assessment including medical history, physical examination and a 12-lead electrocardiogram (ECG) in line with the National Protocol for Emergency Medical Care (LPA version 8.1, June 2016). For the HART‑c study, all regional ambulances were equipped with a Tempus Pro monitor (Philips, Amsterdam, The Netherlands), which streams encrypted patient-specific prehospital data (vital parameters, ECG) to IntelliSpace Corsium (Philips). All acquired data from the clinical assessment and Tempus Pro monitor were transferred to the newly developed digital triage platform.

**Fig. 1:**
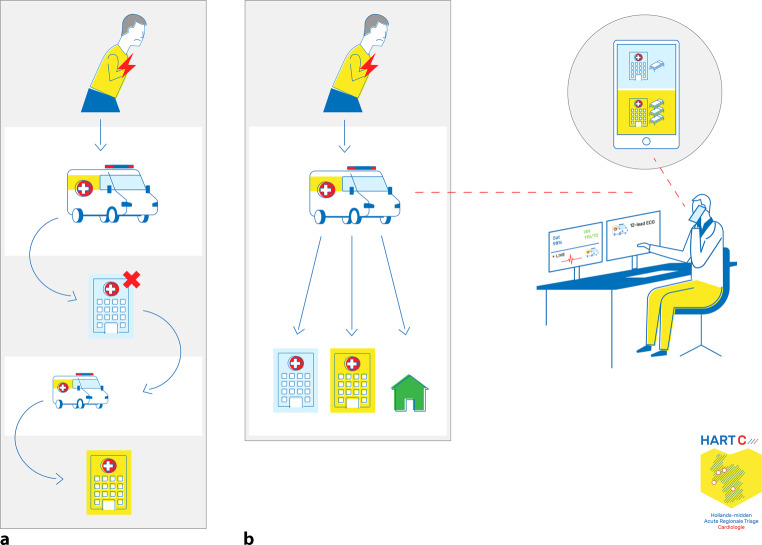
Triage method with **a** standard care and possible interhospital transfers in the case of overcrowded emergency department or nursing ward and **b** novel triage method combining prehospital data with live streaming of electrocardiographic and vital parameters, hospital data such as previous electrocardiograms, medical history and admission capacity as well as expert consultation

After the patient had given informed consent, the nurse paramedic was directly connected with a cardiologist who was on call. On the triage platform, the patient-specific prehospital data were combined with in-hospital data (such as real-time admission capacity for the regional hospitals and previous medical history). The paramedic and cardiologist decided whether transfer to a hospital would be of added value and, if so, which hospital was best suited. The on-call cardiologist noted the decision on the triage platform and a message was automatically sent to the nursing staff of the chosen hospital, thereby immediately informing them of a new referral and updating the hospital’s admission capacity (Fig. 1). Notably, in the control group the nurse paramedic’s decision was based only on prehospital data without consultation with an expert cardiologist.

### Outcome measures

The main objective of the HART‑c study was to investigate whether the triage method could safely increase the number of patients with suspected cardiac symptoms who could stay at home after EMS consultation. For the purposes of this study, safety was defined as the absence of major adverse cardiovascular events (MACE; defined as death or ACS) 30 days after EMS consultation.

Secondary endpoints were the total number of hospital referrals, the total number of interhospital transfers, the time from EMS consultation to hospital arrival, final diagnoses, and patient, general practitioner (GP) and cardiologist satisfaction.

Interhospital transfers were defined as an EMS transfer from one of the three participating hospitals to any other hospital. The final cardiac diagnoses at the ED were assessed using hospital billing data. EMS consultation, ED admission, hospital admission, or GP consultation for any reason within 30 days after EMS consultation were noted. Cardiologists, patients and their GPs rated their satisfaction with the triage method on a scale of 1–10, where 1 was least satisfactory and 10 most satisfactory.

### Statistics

Baseline characteristics were reported as mean and standard deviation (SD) or median and interquartile range (IQR) and compared between control and intervention. The proportions of patients staying at home during the intervention and control periods were compared using binary logistic regression analysis, adjusted for age, sex and month [14]. The total numbers of EMS consultations during the intervention and the control period were compared based on incidence rates based on data on the regional population at the time from Statistics Netherlands (www.cbs.nl), 808,860 and 801,600 in the intervention and control period, respectively, and compared using a chi-squared test. The number of interhospital transfers was compared based on incidence rates from the total number of EMS consultations. The difference in the percentage of final diagnoses per presenting symptom, per ACS diagnosis and in total were evaluated using a chi-squared test. Data were analysed using SPSS Statistics Version 25.0 (IBM Corp., Armonk, NY, USA).

### Ethical conduct

The study complies with the Declaration of Helsinki and was approved by the hospital’s Medical Ethics Committee (P18.213). Patients were requested to provide verbal informed consent before participating in the HART‑c triage study. Patients without a command of spoken Dutch or English were excluded.

## Results

### Baseline

The intervention group comprised 1536 patients (69 ± 15 years, 51.3% male) and the historical cohort control group 1376 patients (68 ± 15 years, 49.9% male). The baseline characteristics of both groups compared well (Tab. 1).

**Table 1:** Baseline characteristics of the control and the intervention group

	Control (*n* = 1376)	Intervention (*n* = 1536)	*p*-value
Age (years)	68 ± 15	69 ± 15	0.181
Sex (male, %)	49.9	51.3	0.637
*Main presenting symptom* (*n, %*)			0.186
Chest pain	733 (53.3%)	880 (57.3%)	
Palpitations	198 (14.4%)	206 (13.4%)	
Dyspnoea	282 (20.5%)	284 (18.5%)	
(Near) syncope	163 (11.8%)	166 (10.8%)	
Sinus rhythm (%)	75.1	77.3	0.182
Breathing frequency (breaths/min)	19 ± 9	19 ± 8	0.153
Oxygen saturation (%)	97 (96–98)	97 (96–98)	0.611
Pulse (beats/min)	90 ± 31	88 ± 29	0.102
Systolic blood pressure (mm Hg)	148 ± 29	150 ± 28	0.220
Diastolic blood pressure (mm Hg)	86 ± 18	86 ± 17	0.987
Distance to hospital (km)	11 ± 8	11 ± 8	0.940

Missing data were excluded. Figures represent mean ± standard deviation or absolute numbers (%)

### Primary objective

**Fig. 2:**
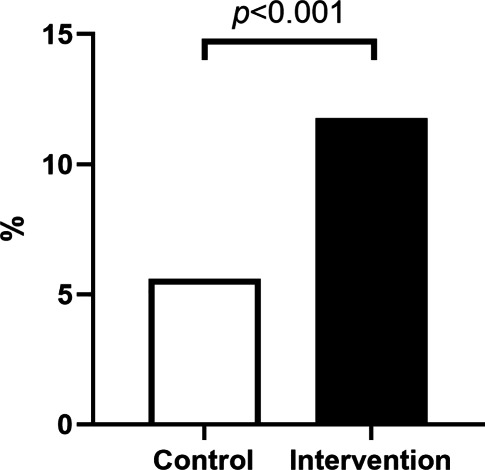
Percentage of patients with cardiac symptoms left at home in the control group (5.9%) and the intervention group (11.8%)

**Fig. 3:**
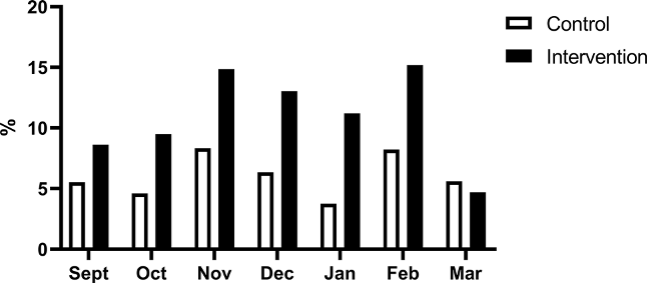
Percentage of patients with cardiac symptoms left at home by the emergency medical services in the control group and in the intervention group per month

In the intervention group, 181 (11.8%) patients could stay at home after EMS consultation, compared to 77 (5.9%) patients in the control group (Fig. 2). The percentage of patients who could stay at home per month is shown in Fig. 3.

Logistic regression showed increased odds of staying at home in the intervention group as compared to the control group: 2.31 (95% confidence interval (CI) 1.74–3.05, *p* < 0.0001). The logistic regression model was adjusted for age, sex and month of presentation (to account for seasonal patterns) (Tab. 2). The most prevalent presenting symptom in patients who could stay at home was chest pain (37.6% in the control and 48.6% in the intervention group), followed by palpitations (36.4% and 27.1%) and dyspnoea (10.4% and 12.2%) (*p* = 0.31).

**Table 2:** Logistical regression model showing the relative risk (*RR*) of staying at home. The final model (in *bold*) was adjusted for age, sex and month of presentation

	RR	95% CI lower limit	95% CI upper limit	*p*-value
**Prehospital triage**	**2.31**	** 1.74**	** 3.05**	**<** **0.001**
Age	0.91	0.87	0.96	**<** **0.001**
Age^2^	1.07	1.03	1.11	**<** **0.001**
Sex (male)	0.70	0.54	0.92	** 0.007**
Month				0.078

‘Age^2^’ is a composite value of (Age × Age) / 100. The *p*-value of ‘Month’ is the likelihood ratio of the contribution of the variable to the logistic regression model

*CI* confidence interval

### Safety

Only 1 of the 181 patients who stayed at home after EMS consultation in the intervention group developed ACS within 30 days after evaluation by the EMS (MACE rate < 1%). No patients died. Five (2.8%) patients were lost to follow-up.

### Secondary endpoints

There were 1536 EMS consultations in the intervention group and 1376 in the control group. The incidence of EMS consultation did not differ between the two groups (190/100,000 vs 172/100,000 inhabitants) with a relative risk of 1.10 (95% CI 0.90–1.36, *p* = 0.344). In the intervention group, the number of interhospital transfers was lower than in the control group (206 vs 173). The incidence of interhospital transfer was significantly lower in the intervention group (173/1355) as compared to the control group (206/1299) with a relative risk of 0.81 (95% CI 0.67–0.97, *p* = 0.023). Time from EMS consultation to hospital arrival increased by 6 min from 37 ± 11 min in the control group to 43 ± 14 min in the intervention group (*p* < 0.001). A triage cardiologist handled an average of 12 calls per day, taking 5–10 min each. The total amount of time a cardiologist spends on prehospital triage is therefore around 1–2 h per day.

In total, 126 (9.2%) control-group patients presenting to the EMS ultimately had an ACS, compared to 127 (8.2%) in the intervention group. All ACS diagnoses are noted in Tab. 3. There were no differences in ACS diagnoses between the control and intervention group (*p* = 0.928). There were no differences in final diagnoses, as shown in Table S1 (Electronic Supplementary Material).

**Table 3:** Overview of final diagnoses of acute coronary syndrome (*ACS*) patients in the control (*n* = 1299) and the intervention group (*n* = 1355) presented to the hospitals

	Control (*n*)	%	Intervention (*n*)	%	*p*-value
ACS	126		127		0.928
STEMI	8	6.3%	8	6.3%	
NSTEMI	85	67.4%	83	65.4%	
Unstable angina	33	26.2%	36	28.3%	

*STEMI *ST-elevation myocardial infarction*, NSTEMI *non-ST-elevation myocardial infarction

### Satisfaction

Patients who could stay at home averaged a satisfaction score of 8.8. The GPs of these patients scored the care given with an average of 7.7. Cardiologists scored each shift, averaging a score of 7.7.

## Discussion

Implementing a novel prehospital triage method for patients visited by the EMS for cardiac symptoms combining live-streamed prehospital data, insight into previous medical history and expert consultation led to a significant increase in the number of patients who could stay at home. The triage method had a MACE rate < 1%; none of these patients died. Furthermore, a decrease in interhospital transfers was achieved. Patients and healthcare workers were very satisfied with the presented triage method. These results may help to relieve the pressure from the currently overcrowded Dutch EDs.

Overcrowding of EDs is a major healthcare challenge [5–8]. Cardiac patients form a large part of all ED consultations, with more than 10% of all ED consultations involving patients experiencing chest pain [1]. However, over 80% of these patients do not suffer from acute cardiovascular disease. To reduce overcrowding, in-hospital triage for rapid risk stratification of chest pain patients has been in use for several years, employing risk scores [9, 15, 16]. Although these result in accurate and fast risk stratification, patients are still presented to and evaluated at the ED, and thus still contribute to overcrowding. Therefore, scientific focus has shifted to improving prehospital triage.

The ARTICA [12] study assesses whether the addition of point-of-care cardiac troponin T (cTnT) measurement is cost-effective in ruling out ACS and leaving patients at home after EMS consultation. The FamouS Triage [17] investigators concluded that it seems feasible and non-inferior to rule out myocardial infarction in prehospital chest pain patients using a modified HEART score at the patient’s home, incorporating only a single cTnT measurement on intravenously acquired blood samples [18]. The PRESTO [19] study seeks to evaluate the diagnostic accuracy of the validated Troponin-only Manchester Acute Coronary Syndromes decision rule to rule out ACS in the prehospital environment. This could allow paramedics to rule out ACS in chest pain patients in the very low risk group and avoid the need for transport to the ED.

The present HART‑c study differs from the aforementioned studies as regards the patients included, the triage method and its main objective. Firstly and uniquely, the HART‑c study does not limit inclusion to chest pain patients, but also includes patients with other cardiac symptoms. Thus, the HART‑c study could be of benefit to a larger cohort of patients and, for this reason, could be of greater value in preventing overcrowding of EDs and hospitals. Since the method is not solely focused on chest pain patients, it is easier to recreate, adjust and implement for other medical specialties. Second, the HART‑c study is unique in its triage method. The ARTICA, FamouS and PRESTO studies rely solely on prehospital data, whereas the HART‑c study is the first study to combine prehospital and hospital data. Furthermore, the HART‑c study includes expert on-scene consultation by having a cardiologist available for the nurse paramedic. Lastly, this is the first study to publish its results regarding safely leaving cardiac patients at home after EMS assessment. Appropriate selection of patients at (very) low risk for MACE, who could therefore safely stay at home following EMS consultation, could contribute substantially to providing overcrowded EDs and hospitals with much-needed relief. Of utmost importance, patients who can safely stay at home after EMS consultation are spared the (unnecessary) strain and stress of an ED visit.

The HART‑c study has some limitations. First of all, this is not a randomised controlled trial, so selection bias could influence its results and therefore these results should be seen as promising and not definitive. A randomised controlled trial or a study with a stepped wedge design should be conducted in the future to confirm the results presented in the current study. Another limitation is the number of patients lost to follow-up when not transferred to an ED. Unfortunately, not all these patients (or their GPs) could be contacted after 30 days, as some patients (tourists or homeless) did not have a GP or in some cases phone numbers were not noted correctly. Therefore, the true MACE rate could be slightly higher than 1%. Furthermore, the MACE rate might have been this low due to the broad inclusion criteria: ‘symptoms of possible cardiac origin’. Chest pain patients with possible ACS have a higher MACE rate than, for example, patients with palpitations or dyspnoea. The intervention was planned for 1 year. However, in March 2020 COVID-19 struck the Netherlands, which had a huge impact on acute and non-acute (cardiac) care. As patients might have hesitated to contact the GP, EMS or hospitals this would have introduced too much bias in the study, affecting the comparability between the intervention and the (historical) control group. Therefore, the study was closed in March 2020.

In conclusion, the HART‑c study evaluated a novel triage method combining prehospital live-streamed EMS data, insight into previous medical records, real-time hospital admission capacity with consultation of an expert cardiologist. The achieved increase in patients who could safely stay at home after EMS consultation and the reduction in interhospital transfers could help take substantial pressure off the currently overloaded healthcare system. Furthermore, the presented triage method is adjustable and easily implementable for other medical specialties to further reduce overcrowding.

## Supplementary Information

Table S1. Overview of diagnoses per presenting symptom of patients presented to the hospitals for control—(n = 1299) and intervention group (n = 1355).

## References

[1] Bhuiya FA, Pitts SR, McCaig LF. Emergency department visits for chest pain and abdominal pain: United States, 1999-2008. NCHS Data Brief. 2010:1–8. PMID: 20854746. 20854746

[2] Goodacre S, Thokala P, Carroll C (2013). Systematic review, meta-analysis and economic modelling of diagnostic strategies for suspected acute coronary syndrome. Health Technol Assess.

[3] Gorenberg M, Marmor A, Rotstein H (2005). Detection of chest pain of non-cardiac origin at the emergency room by a&nbsp;new non-invasive device avoiding unnecessary admission to hospital. Emerg Med J.

[4] Mol KA, Rahel BM, Meeder JG (2016). Delays in the treatment of patients with acute coronary syndrome: focus on pre-hospital delays and non-ST-elevated myocardial infarction. Int J Cardiol.

[5] Boyle A, Beniuk K, Higginson I (2012). Emergency department crowding: time for interventions and policy evaluations. Emerg Med Int.

[6] American College of Emergency PhysiciansCrowding (2013). Policy statement. Ann Emerg Med.

[7] Sun BC, Hsia RY, Weiss RE (2013). Effect of emergency department crowding on outcomes of admitted patients. Ann Emerg Med.

[8] Rasouli HR, Esfahani AA, Nobakht M (2019). Outcomes of crowding in emergency departments; a&nbsp;systematic review. Arch Acad Emerg Med.

[9] Six AJ, Backus BE, Kelder JC (2008). Chest pain in the emergency room: value of the HEART score. Neth Heart J.

[10] Backus BE, Six AJ, Kelder JC (2010). Chest pain in the emergency room: a&nbsp;multicenter validation of the HEART Score. Crit Pathw Cardiol.

[11] van Dongen DN, Tolsma RT, Fokkert MJ (2020). Referral decisions based on a&nbsp;prehospital HEART score in suspected non-ST-elevation acute coronary syndrome: design of the FamouS Triage 3&nbsp;study. Future Cardiol.

[12] Aarts GWA, Camaro C, van Geuns RJ (2020). Acute rule-out of non-ST-segment elevation acute coronary syndrome in the (pre)hospital setting by HEART score assessment and a&nbsp;single point-of-care troponin: rationale and design of the ARTICA randomised trial. BMJ Open.

[13] de Koning E, Biersteker TE, Beeres S (2021). Prehospital triage of patients with acute cardiac complaints: study protocol of HART-c, a&nbsp;multicentre prospective study. Bmj Open.

[14] Kurihara O, Takano M, Yamamoto E (2020). Seasonal Variations in the pathogenesis of acute coronary syndromes. J Am Heart Assoc.

[15] Boyle RSJ, Body R (2021). The diagnostic accuracy of the Emergency Department Assessment of Chest Pain (EDACS) score: a&nbsp;systematic review and meta-analysis. Ann Emerg Med.

[16] Body R, Carlton E, Sperrin M (2017). Troponin-only Manchester Acute Coronary Syndromes (T-MACS) decision aid: single biomarker re-derivation and external validation in three cohorts. Emerg Med J.

[17] Ishak M, Ali D, Fokkert MJ (2018). Fast assessment and management of chest pain patients without ST-elevation in the pre-hospital gateway (FamouS Triage): ruling out a&nbsp;myocardial infarction at home with the modified HEART score. Eur Heart J Acute Cardiovasc Care.

[18] Tolsma RT, Fokkert MJ, van Dongen DN (2022). Referral decisions based on a&nbsp;pre-hospital HEART score in suspected non-ST-elevation acute coronary syndrome: final results of the FamouS Triage study. Eur Heart J Acute Cardiovasc Care.

[19] Alghamdi A, Cook E, Carlton E (2019). PRe-hospital Evaluation of Sensitive TrOponin (PRESTO) study: multicentre prospective diagnostic accuracy study protocol. BMJ Open.

