# Supplementary material for: Improved prehospital triage for acute cardiac care: results from HART-c, a multicentre prospective study
Source: Neth Heart J. 2023 Mar 29;31(5):202–9. doi: 10.1007/s12471-023-01766-3 (PMC10050817; doi:10.1007/s12471-023-01766-3)
Supplement: Supplementary file 1 — Table S1. Overview of diagnoses per presenting symptom of patients presented to the hospitals for control—(n = 1299) and intervention group (n = 1355). [file 12471_2023_1766_MOESM1_ESM.docx]

*Table S1. Overview of diagnoses per presenting symptom of patients presented to the hospitals for control - (n=1299) and intervention group (n=1355).*

|  | Control (n) | % | Intervention (n) | % | p-value |
| --- | --- | --- | --- | --- | --- |
| Chest pain | **704** |  | **792** |  | **0,059** |
| Acute coronary syndrome | 110 | 15,6% | 111 | 14,0% |  |
| Stable angina | 33 | 4,7% | 25 | 3,2% |  |
| Supraventricular tachycardia | 37 | 5,3% | 40 | 5,1% |  |
| Chest pain of unknown cause | 364 | 51,7% | 434 | 54,8% |  |
| Other cardiac | 61 | 8,7% | 48 | 6,1% |  |
| Pulmonary embolism | 3 | 0,4% | 5 | 0,6% |  |
| Other pulmonary pathology | 5 | 0,7% | 4 | 0,5% |  |
| Vascular pathology | 6 | 0,9% | 3 | 0,4% |  |
| Abdominal pathology | 3 | 0,4% | 6 | 0,8% |  |
| Hypertension | 24 | 3,4% | 25 | 3,2% |  |
| Miscellaneous | 25 | 3,6% | 50 | 6.3% |  |
| Diagnosis unknown | 33 | 4,7% | 41 | 5,2% |  |
| Palpitations | **170** |  | **157** |  | **0,770** |
| Acute coronary syndrome | 4 | 2,4% | 5 | 3,2% |  |
| Supraventricular tachycardia | 68 | 40,0% | 59 | 37,6% |  |
| Ventricular tachycardia | 6 | 3,5% | 8 | 5,1% |  |
| No arrythmia | 62 | 36.5% | 57 | 36.3% |  |
| Other cardiac | 18 | 10,6% | 21 | 13,4% |  |
| Pulmonary embolism | 1 | 0,6% | 0 | 0,0% |  |
| Other pulmonary pathology | 3 | 1,8% | 0 | 0,0% |  |
| Vascular pathology | 2 | 1,2% | 2 | 1,3% |  |
| Abdominal pathology | 1 | 0,6% | 0 | 0,0% |  |
| Miscellaneous | 3 | 1,8% | 3 | 1,9% |  |
| Diagnosis unknown | 5 | 2,9% | 5 | 3,2% |  |
| Dyspnea | **274** |  | **262** |  | **0,650** |
| Acute coronary syndrome | 10 | 3,6% | 8 | 3,1% |  |
| Heart failure | 74 | 27,0% | 68 | 26,0% |  |
| Dyspnea of unknown cause | 56 | 20,4% | 45 | 17,2% |  |
| Other cardiac | 30 | 10,9% | 36 | 13,7% |  |
| Pulmonary embolism | 7 | 2,6% | 3 | 1,1% |  |
| Other pulmonary pathology | 30 | 10,9% | 33 | 12,6% |  |
| Vascular pathology | 1 | 0,4% | 4 | 1,5% |  |
| Miscellaneous | 45 | 16,4% | 46 | 17,6% |  |
| Diagnosis unknown | 21 | 7,7% | 19 | 7,3% |  |
| Other | **151** |  | **144** |  | **0,273** |
| Acute coronary syndrome | 2 | 1,3% | 2 | 1,4% |  |
| Supraventricular tachycardia | 12 | 7,9% | 8 | 5,6% |  |
| Ventricular tachycardia | 2 | 1,3% | 7 | 4,9% |  |
| Cardiac syncope | 31 | 20,5% | 33 | 22,9% |  |
| Symptom of unknown cause | 44 | 29,1% | 30 | 20,8% |  |
| Other cardiac | 5 | 3,3% | 9 | 6,3% |  |
| Miscellaneous | 47 | 31.1% | 43 | 29.9% |  |
| Diagnosis unknown | 8 | 5,3% | 12 | 8,3% |  |
